# Supplementary material for: Pyk2/FAK Signaling Is Upregulated in Recurrent Glioblastoma Tumors in a C57BL/6/GL261 Glioma Implantation Model
Source: Int J Mol Sci. 2023 Aug 30;24(17):13467. doi: 10.3390/ijms241713467 (PMC10487692; doi:10.3390/ijms241713467)
Supplement: Supplementary file 1 [file ijms-24-13467-s001.zip › Supplementary Figures captions.pdf]

Figure S1: PTK2 (FAK) and PTK2B (Pyk2) mRNA expression (Log2 Z-score) in the TCGA glioblastoma RNA-Sequence dataset (obtained from the R2 Genomic Analysis Visualization Platform) indicated no significant difference in Pyk2 and FAK gene expression between newly diagnosed (no, 496 specimens) and recurrent (yes, 15 specimens) GBM tumors.

Figure S2: Whole-membrane Western blot images and loading controls in support of Figure 1.

Figure S3: Whole-membrane Western blot images and loading controls in support of Figures 3A and 4A.

Figure S4: H&E staining for brain sections encompassing individual recurrent tumors from both the vehicle and PF-562271 treatment groups in support of Figure 5A. The tumor resection was performed 14 days after tumor implantation. PF-562271 or vehicle were given to animals 2 days before and, then, after the tumor resection, 50 mg/kg/day, every day. Data are presented for tumors regrown 14 days after the surgical resection.
