# Supplementary figures and images for: Pyk2/FAK Signaling Is Upregulated in Recurrent Glioblastoma Tumors in a C57BL/6/GL261 Glioma Implantation Model
Source: Int J Mol Sci. 2023 Aug 30;24(17):13467. doi: 10.3390/ijms241713467 (PMC10487692; doi:10.3390/ijms241713467)

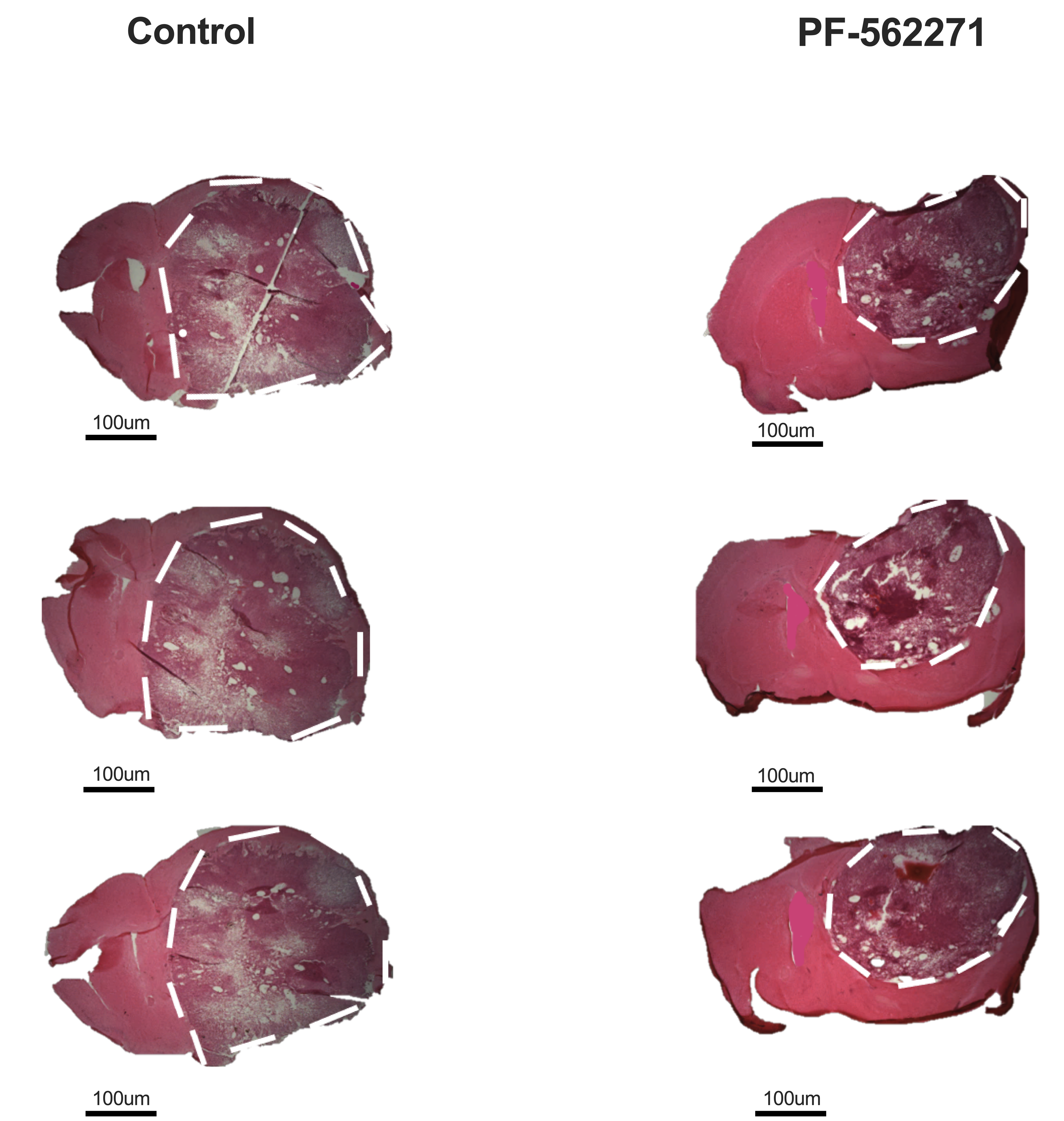

Supplement: Supplementary file 1 [file ijms-24-13467-s001.zip › Supplementary S4 .tiff]

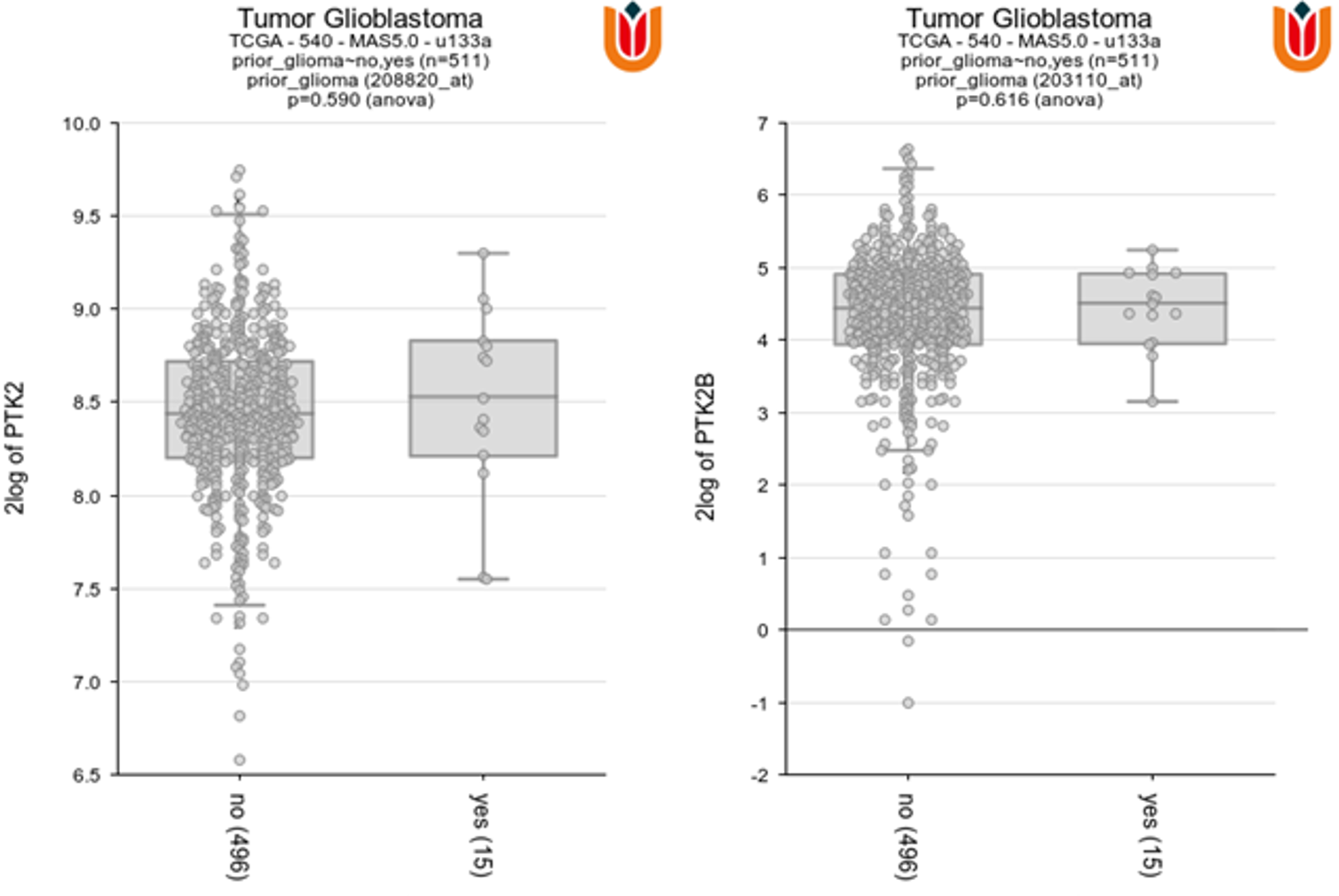

Supplement: Supplementary file 1 [file ijms-24-13467-s001.zip › Supplementary Figure S1.tif]

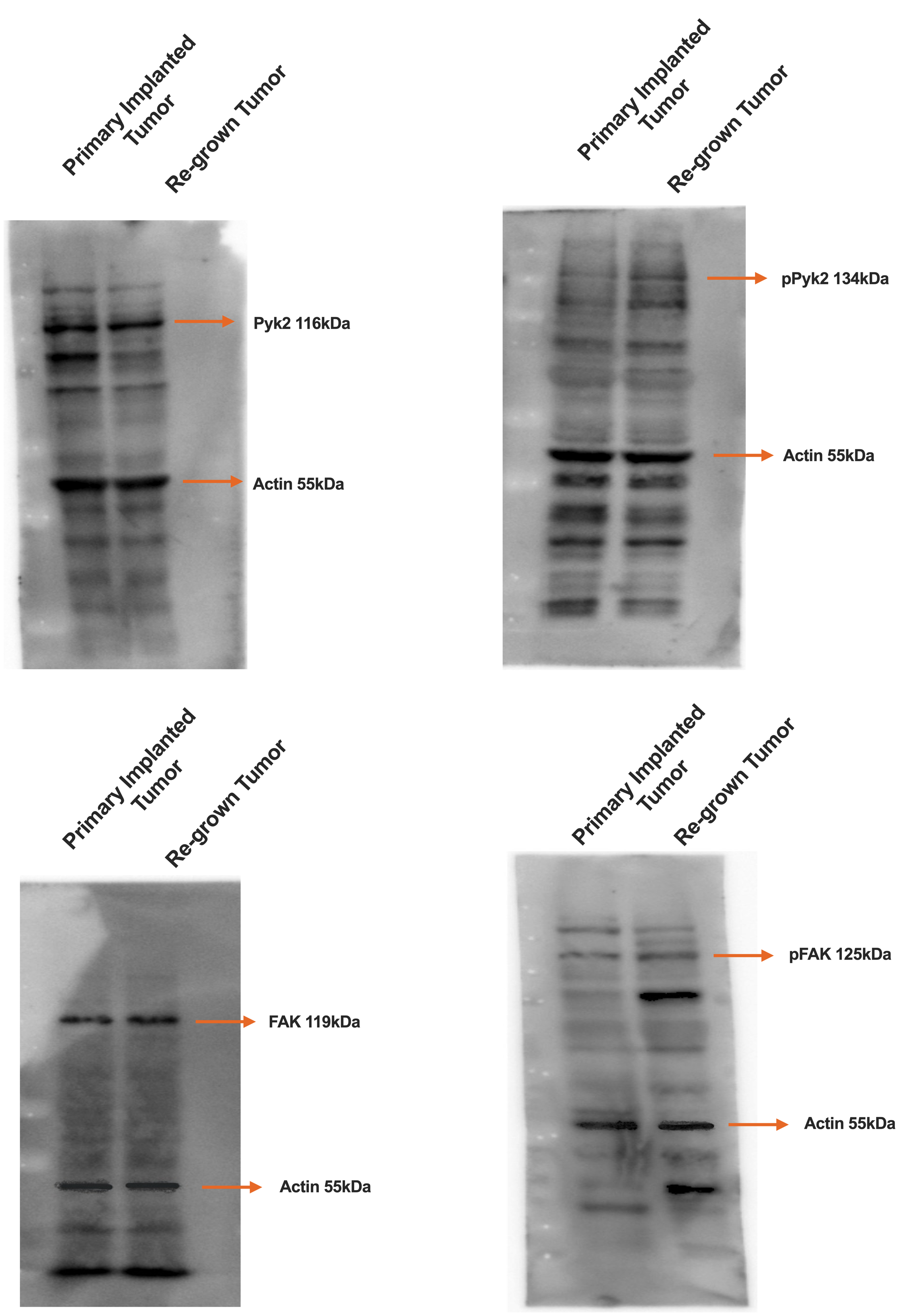

Supplement: Supplementary file 1 [file ijms-24-13467-s001.zip › Supplementary Figure S2.tiff]

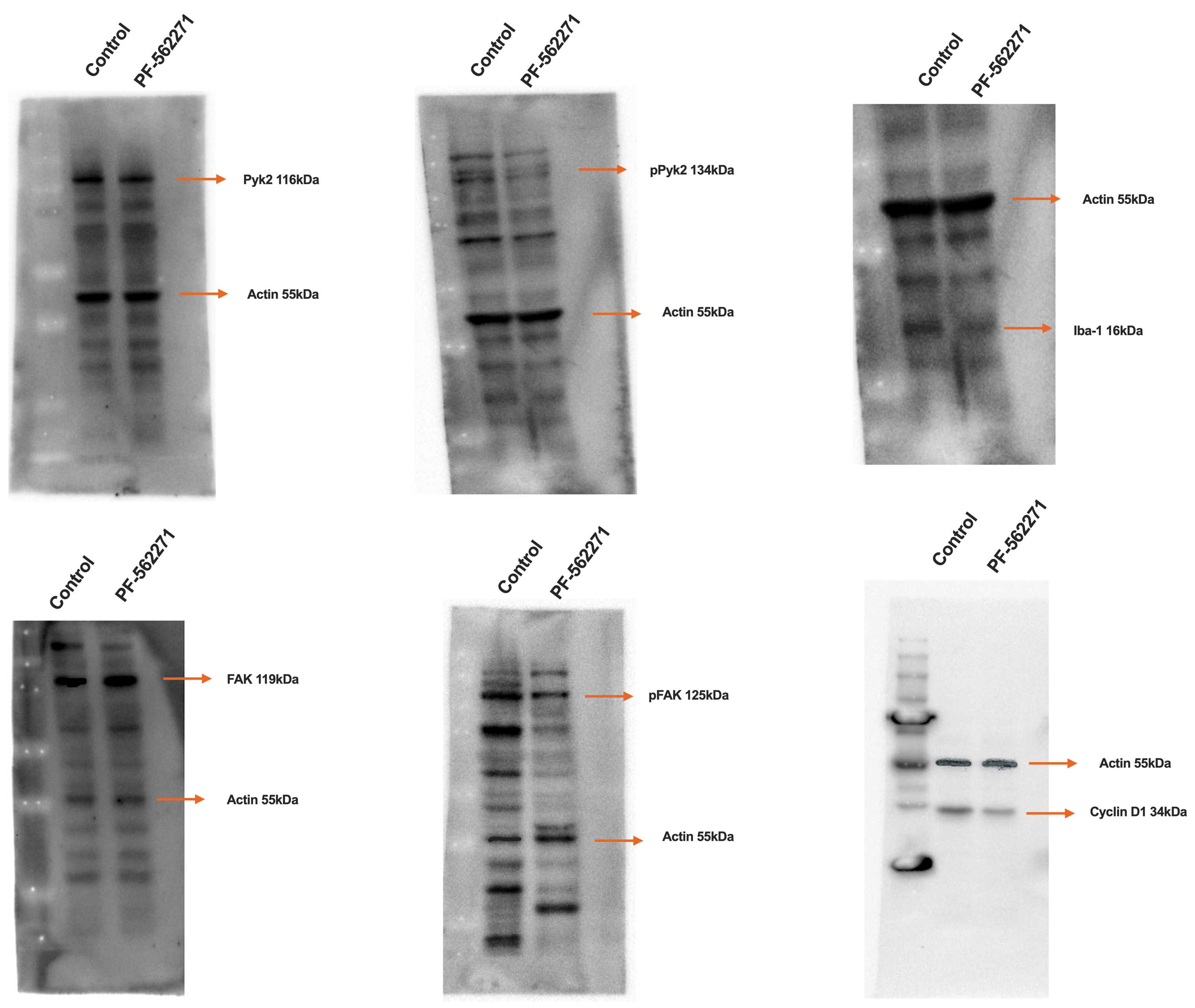

Supplement: Supplementary file 1 [file ijms-24-13467-s001.zip › Supplementary S3.tiff]
